# Infections Are a Very Dangerous Affair: Enterobiasis and Death

**DOI:** 10.3390/healthcare9121641

**Published:** 2021-11-27

**Authors:** Gelsomina Mansueto, Mariavictoria De Simone, Paola Ciamarra, Emanuele Capasso, Alessandro Feola, Carlo Pietro Campobasso

**Affiliations:** 1 Department of Advanced Medical and Surgical Sciences (DAMSS), University of Campania Luigi Vanvitelli, 80138 Naples, Italy; gelsomina.mansueto@unicampania.it; 2 Clinical Department of Laboratory Services and Public Health-Legal Medicine Unit, University of Campania Luigi Vanvitelli, via Luciano Armanni 5, 80138 Naples, Italy; 3 Department of Experimental Medicine, University of Campania Luigi Vanvitelli, 80138 Naples, Italy; paola.ciamarra@studenti.unicampania.it (P.C.); alessandro.feola@unicampania.it (A.F.); carlopietro.campobasso@unicampania.it (C.P.C.); 4 Department of Advanced Biomedical Sciences, University of Naples Federico II, 80138 Naples, Italy; emanuele.capasso@unina.it

**Keywords:** enterobiasis, oxyuriasis, forensic autopsy, death, histology

## Abstract

Background: Enterobiasis or oxyuriasis from Enterobius vermicularis is an infection usually localized in the large bowel and cecum. Generally, the symptoms are characterized by anal itching, and intestinal or nervous disorders. Rarely, it is responsible for death. Methods: A forensic autopsy of a 52-year-old white male inmate who died 5 days after hospitalization was performed. Histological and toxicological analyses were also performed. Results: The death occurred by localization of Enterobius vermicularis in the duodenum and in the proximal ileum, with intestinal haemorrhage, inflammation, and peritonitis documented by histological examination. Conclusion: This is a common infectious disease, and can rarely occur with a fatal outcome, even in advanced populations. The lack of knowledge related to the rarity of death from enterobiasis disease can determine a dangerous concern.

## 1. Introduction

Gastrointestinal infections are a less common cause of sudden death compared to other conditions such as cardiovascular diseases, but they are equally important [1,2]. Among gastrointestinal diseases, Enterobius vermicularis (EV) infections represent an underestimated rarity to consider. The EV or pinworn is a nematod, one of the most common intestinal parasites found in humans, which generally colonize the large bowel and the cecum. The infection caused by EV is known as enterobiasis or oxyuriasis, and affects about 200 million people around the world, mainly children, with a symptomatology characterized by anal itching and intestinal disorders. However, the involvement of the gastrointestinal tract can be severe and cause death [1,2,3,4,5,6,7,8,9]. The mortality rate due to EV is not significant, but infections caused by intestinal worms can be a significant public health and economic concern in countries where these infections remain endemic. This is a common infectious disease in temperate regions, and rarely occurs with a fatal outcome, even in advanced disease populations. Although this agent is known, but also controversial, for its role in the etiology of acute [10], less known is its causal role in some deaths. The lack of knowledge related to the rarity of death from enterobiasis disease can determine a dangerous situation.

We report a case of death with forensic autopsy, in which EV in the duodenum and in the proximal ileum was found histologically. Such a death is rare, but this report shows that it can occur.

## 2. Case Presentation

Case History: A 52-year-old white male inmate with a history of non-steroidal anti-inflammatory drugs (NSAIDs) therapy and enalapril therapy for hypertension was admitted to the emergency room for repeated lipothymia in the absence of sweating, with hematemesis from the previous evening and melaena from three days before. The patient was hemodynamically unstable with acute anemia. The hemoglobin value upon admission was 6g/dL, while the procalcitonin in the blood was not evaluated. Therefore, a computed tomography (CT) scan of the abdomen was performed, which revealed a narrow lumen of the second portion of the duodenum; furthermore, the esophagus-gastro-duodenoscopy (EGDS) examination revealed multiple sub-centimeter lymph node formations in the stomach with normodistended walls due to insufflation, and fundus and gastric bodies occupied by food residues and clots; at the level of the first duodenum, there was an ulcerated lesion covered by a large clot. After a worsening of the condition, the patient was transferred to Intensive Care, was intubated and underwent therapy to restore hemodynamic balance. On the fifth day, the hemodynamics were unstable, and the anemia persisted. An emergency gastroscopy was performed in resuscitation, which revealed the absence of blood in the esophagus, stomach, and duodenum, and ulcerative lesion of the duodenal bulb with circumferential extension to the intestinal wall. Conditions precipitated due to common complications of hypovolemia. Hemorrhagic shock and peritonitis due to enterobiasis were assessed as causes of death. After 72 h, an autopsy was performed in accordance with the recommendations on the harmonization of forensic autopsy rules of the Committee of Ministers of the Council of Europe (1999) and according to the commonly accepted criteria for sudden cardiac death (SCD). Femoral blood was analyzed for alcohol (ethanol) and volatiles by head-space gas chromatography coupled with a flame ionization detector (GC/HS-FID). All post-mortem specimens were screened for the presence of the main different classes of drugs (pharmaceuticals and illegal drugs), using immunological or chromatographic methods as appropriate. A systematic toxicological analysis (STA) was performed by the LC-MS/MS system (API 3200 triple quadrupole ABI-SCIEX) in multiple reaction monitoring (MRM) mode.

## 3. Results

### 3.1. Autopsy Findings: Macroscopic Findings

**Figure 1:**
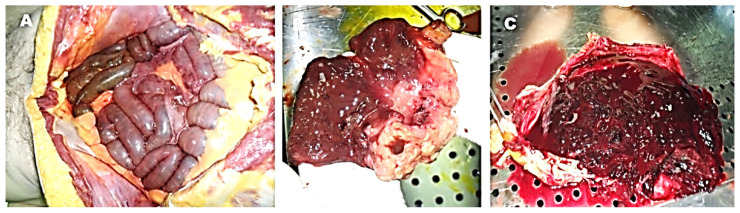
Autopsy findings. No fluid in the peritoneal cavity between the intestinal loops and no of macroscopic evident perforations (**A**). Macroscopic observation of the duodenum showed ulcerative lesion of the second portion with narrowing (**B**). The lumen of stomach was full of abundant blood clots (**C**).

The autopsy showed some thin blood-stained fluid in the peritoneal cavity between the intestinal loops at the duodenum and the proximal ileum in the absence of macroscopic evident perforations (Figure 1A). We did not detect any pathology in the other thoracic, abdominal, or pelvic organs, or the brain. Macroscopic observation of the duodenum showed ulcerative lesion of the second portion with narrowing of the lumen and stomach replenishing with coarse food material mixed with abundant blood clots and blood (Figure 1B–C).

### 3.2. Toxicological Analysis

The toxicological analysis showed no evidence of drugs or alcohol.

### 3.3. Microscopic Findings. Histology

**Figure 2:**
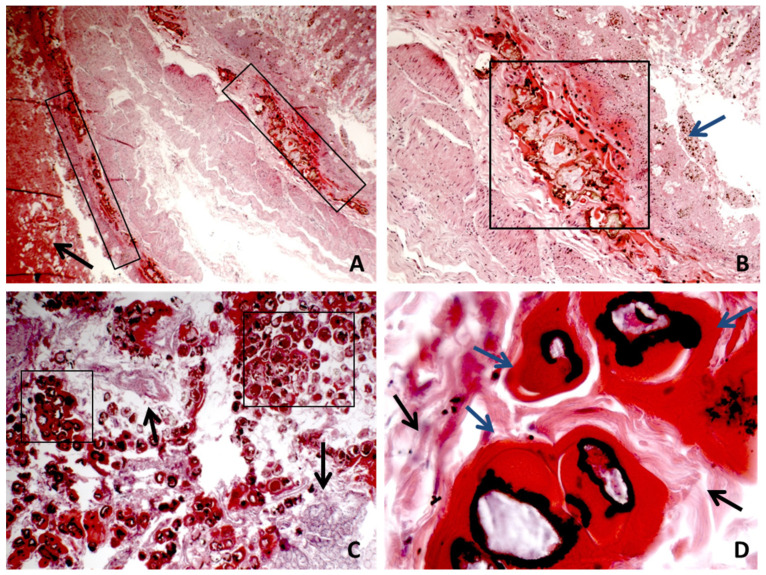
(**A**,**B**) The panoramic of wall interruption, with hemorrhage, necrosis, inflammation, and Enterobius vermicularis accumulation in different bowel sections. The black arrow indicates large areas of bleeding. The black box shows the presence of cross-sections of EV with interruption of the muscolaris mucosa. The blue arrow indicates the mucosa that is no longer recognizable cytologically due to destruction and necrosis ((**A**) H&E ×4 magnification; (**B**) H&E ×10 magnification). (**C**) Area with a more evident presence of EV. The black box shows cross sections of EV; the black arrow indicates the residues of the intestinal wall fragmentation (H&E ×10 magnification). (**D**) High magnification of the transverse sections of the EV body; the black arrow indicates the residues of the intestinal wall fragmentation and the blue arrow indicate the EV body (H&E ×40).

For each organ, sampling was carried out. After fixation in 10% neutral buffered formalin and paraffin embedding, sections for hematoxylin−eosin (E&E) staining for morphological diagnosis were performed. The histology of the duodenum and proximal ileum tract showed diffuse necrosis with multiple areas where trans-mural hemorrhage, granulocytic inflammation, and EV were detected (Figure 2). In particular, a continuous interruption of the sub-mucosal tunic due to the presence of EV was observed (Figure 2). Congestion, oedema, and inflammation were detected in the other organs and in the liver.

## 4. Discussion

**Figure 3:**
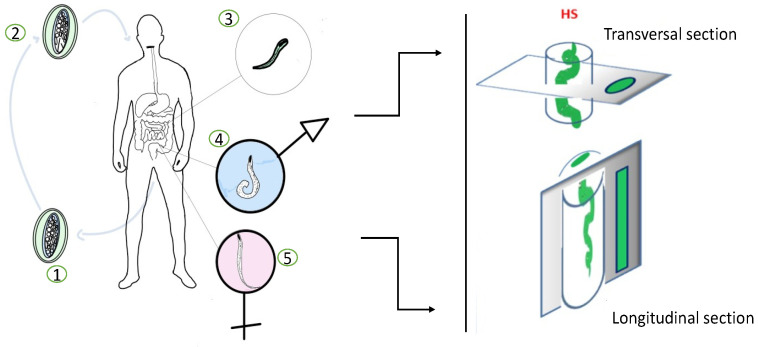
Life cycle of EV in human and intestinal sampling for histological examination. (1) Eggs on perianal folds. Larvae inside the eggs mature within 4 to 6 h. (2) Embryonated eggs ingested by human. (3) Larvae hatch in small intestine. (4) Adults in lumen of cecum. (5) Gravid female migrates to perianal region at night to lay eggs. HS—histological sections.

Enterobiasis or oxyuriasis is an intestinal parasitosis that mainly affects children, with predominantly anal itching and intestinal symptoms. After the ingestion of infective eggs, larvae hatch in the duodenum and descend into the crypts of the duodenum and the ileum where they develop and mutate into adult worms. In the beginning, larvae adhere to the mucosal surfaces of the small intestine, where they develop to sexually mature adult form and establish a chronic infection causing mucosal inflammation and ulceration. Then, the larvae can also penetrate the deeper layers of the intestinal wall where they establish chronic inflammation, destroying the tissue layers. Commonly, adult individuals settle in the colon from where pregnant females migrate to the perianal area at night, while the host is sleeping, to lay eggs. In fact, a diagnosis of enterobiasis in the living can be performed by applying a transparent adhesive tape in the perianal area immediately after waking up, followed by microscopic analysis. A reliable diagnosis of enterobiasis is also based on a correct intestinal sampling. Multiple samples of the intestinal wall must be taken at random in order to find the pinworms inside. In histological samples, the thick cuticles and internal organs of the worms can be easily recognizable within the intestinal wall, from the mucosal epithelium to the serosa, according to the transverse and longitudinal sections of paraffin inclusions (Figure 3).

Sometimes, although very rarely, EV can also migrate into the other tracts, such as the urinary bladder, peritoneum, kidneys, liver, lungs, and other organs. Generally, it is considered that EV is a non-invasive parasite, which cannot penetrate a healthy intestinal mucous membrane. However, there is increasing evidence that EV infection may be the cause of ileal inflammation. By causing mucosal inflammation and ulceration, pinworms damage the integrity of the bowel mucosa and can then penetrate the deeper layers of the bowel wall or lead to intestinal perforation and hemorrhage [11,12,13,14]. Narrowing of the intestinal lumen is commonly the result of mucosal inflammation and ulceration produced by the parasitosis, which can easily be appreciated in CT scans as well as at autopsy. It seems that unexplained gastrointestinal symptoms are quite common in adults who recall abuse as children. However, in this case study, no evidence of child abuse was found in the past history of the victim [15]. Physicians should consider the possibility of infection by EV during the examination of the genitalia because of child infected with EV can occasionally scratch his/her perianal region and make a reddening in the anal region mimicking sexual abuse [16]. In our case, very rare complications of EV infection occurred, represented by perforation, hemorrhage, and destructive inflammation of the intestinal wall that were difficult to treat due to the lack typical symptoms and prompt diagnosis. Intestinal bleeding and inflammation (with the release of chemical mediators) caused lethal peritonitis and hemorrhagic shock.

This case is interesting because it offers a reflection in the framing of an inmate with a history of drugs associated with repeated events of lipothymia, hematemesis, and melaena caused by intestinal parasitosis. Unfortunately, there was not enough time to diagnose the EV infections due to the rapid worsening of the patient’s condition. The instrumental investigations were able to identify the ulcerative complications that may be common to different diseases, such as the side effects of drug medications. A CT of the abdomen, EGDS, and gastroscopy showed the absence of blood in the oesophagus, stomach, and duodenum, along with ulcerative lesions of the duodenal bulb with circumferential extension to the intestinal wall. However, the diagnosis of EV infections is generally made on the identification of the eggs in the anal region or of the pinworm after intestinal biopsy. Therefore, intestinal parasitosis should be considered in the differential diagnosis of clinical syndromes like gastrointestinal haemorrhages.

## 5. Conclusions

This case study presents the worst consequences of enterobiasis in a young adult. A large number of human pinworms were observed histologically in the duodenum and ileum wall, surrounded by inflammatory cell infiltration, bowel hemorrhage, and peritonitis. Therefore, a correct differential diagnosis between enterobiasis and gastrointestinal ulcer hemorrhages from other causes can be useful for correct treatment of patients.

## Data Availability

The data presented in this study are available on request from the corresponding author. The data are not publicly available due to privacy restriction.
